# Investigation of PZT Materials for Reliable Piezostack Deformable Mirror with Modular Design

**DOI:** 10.3390/mi14112004

**Published:** 2023-10-28

**Authors:** Vladimir Toporovsky, Vadim Samarkin, Alexis Kudryashov, Ilya Galaktionov, Alexander Panich, Anatoliy Malykhin

**Affiliations:** 1 Sadovsky Institute of Geosphere Dynamics, Russian Academy of Sciences, Leninskiy Pr. 38/1, Moscow 119334, Russia; samarkin@nightn.ru (V.S.); kud@activeoptics.ru (A.K.); galaktionov@activeoptics.ru (I.G.); 2 Department of Physics, Moscow Polytechnic University, Bolshaya Semenovskaya Str. 38, Moscow 107023, Russia; 3 STCB ‘Piezopribor’, Institute for Advanced Technologies and Piezotechnics SFEDU, Milchakova Str. 10, Rostov-on-Don 344090, Russia

**Keywords:** adaptive optics, PZT materials, relaxor ferroelectrics, wavefront control, deformable mirrors, multilayer actuator

## Abstract

This article presents a study of the electrophysical properties of a piezoceramic material for use in adaptive optics. The key characteristics that may be important for the manufacturing of piezoelectric deformable mirrors are the following: piezoelectric constants (d_31_, d_33_, d_15_), capacitance, elastic compliance values s for different crystal directions, and the dielectric loss tangent (tgδ). Based on PZT ceramics, the PKP-12 material was developed with high values of the dielectric constant, piezoelectric modulus, and electromechanical coupling coefficients. The deformable mirror control elements are made from the resulting material—piezoceramic combs with five individual actuators in a row. In this case, the stroke of the actuator is in the range of 4.1–4.3 microns and the capacitance of the actuator is about 12 nF.

## 1. Introduction

Since the 1880s, studies of the properties of ferroelectrics have been carried out [1]. A special place in this series is occupied by piezoelectric ceramics, since such materials are capable of converting mechanical load into electrical energy and vice versa, and they could be used in various fields, such as smart sensing [2], energy harvesting [3], and as actuators for different applications [4,5]. The use of the piezoceramic devices as actuators can mainly be divided into three main areas: vibration suppressors [6], motors [7], and positioners [8]. The high reliability and positioning accuracy of such devices make it possible to use them in precision optical devices such as: fast steering mirrors [9,10], opto-mechanical adjustment systems (stepper motors [11], micro- and nanopositioning stages [12], etc. [13]), and piezoelectric wavefront correctors (bimorph [14,15,16,17,18,19,20,21], piezostack [22,23,24,25,26,27,28,29,30,31], etc. [32,33]) in various areas [34,35,36,37,38,39,40,41,42,43,44,45]. Considering the latter, it should be noted that piezomaterials manufactured for deformable mirrors [46,47,48,49,50] or tip-tilt stages [51] are subject to special requirements: high sensitivity to the applied voltages, reasonable stroke and blocking force, and the lowest possible electrical capacitance [52] to simplify the control electronics. The development of the multilayer stacks allowed for the generation of a large stroke at relatively low voltages (0…+120–300 V). Additional mechanisms can also be equipped to increase the displacement of the actuators to hundreds of microns: lever- [53], bridge- [54], and four-bar linkage [55] or Scott–Russell-type designs [56,57].

In the manufacturing of the piezoelectric deformable mirrors, solid solutions based on lead zirconate titanate (PZT) are used as a material for creating control elements due to their high electrical properties (piezoceramic coefficients d_31_, d_33_), dielectric loss tangent, and blocking force [58,59,60]. Piezoceramic coefficients correspond to the deformable mirror stroke for compensation of the wavefront aberrations with the large amplitude. The dielectric loss tangent value impacts the ability of the wavefront corrector to mitigate influence of the aberrations dynamically changed with high speed. The blocking force parameter characterizes the maximum value of the force generated by a piezostack that is locked by a rigid, unyielding hold [61,62]. In addition, when creating piezostack deformable mirrors, a problem arises when increasing the spatial resolution of the control elements and, at the same time, obtaining a reliable design [63].

The paper is organized as follows. The Section 2 discusses the main parameters and properties of the piezomaterial for the manufacturing of the deformable mirror actuators. Based on such studies, piezoceramic modules were fabricated and their characteristics are discussed in Section 3. The conclusions are presented in Section 4.

## 2. Materials and Methods

### 2.1. Piezoelectric Effect and Material Properties

The piezoelectric effect could be explained by the property of the elementary cell of the material structure. In the elementary cell—the smallest symmetrical unit of material, from which, by repeating it many times, a microscopic crystal can be obtained—arises the non-symmetry of the center of the symmetry [64].

**Figure 1:**
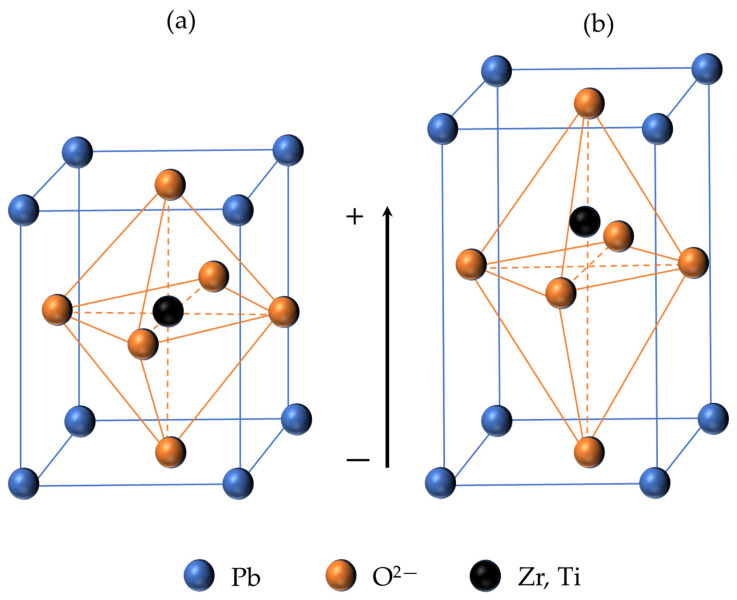
Behavior of the elementary cell of the PZT crystal above the Curie temperature (**a**) and below Curie temperature (**b**).

The elementary cell at temperatures above the critical one (Curie temperature [65]) has a cubic crystal lattice. If the temperature is below Curie temperature, then the elementary cell is distorted tetragonally towards one of the edges. As a result, the distances between positively and negatively charged ions also change (Figure 1). The displacement of ions from their original position leads to a separation of the centers of gravity of the charges within the cell, so that an electric dipole moment is formed. According to the energy conditions, the dipoles of neighboring elementary cells of the crystal are ordered by regions in the same direction, forming the so-called domains.

Mechanical compression or tension acting on a piezoelectric plate parallel to the direction of polarization leads to deformation of all elementary cells. In this case, the centers of gravity of the charges are mutually displaced inside the elementary cells, which are now predominantly parallel, and as a result, a charge is obtained on the surface.

The piezoelectric effect could be described in two main ways: direct and inverse phenomena. For sensing or harvesting applications, the piezoelectric material generates an electric signal under mechanical pressure that is expressed with an electrical displacement D of the material—this is the direct piezoelectric effect. At the same time, such materials could be exploited for actuation applications, however, it generates mechanical strain X under an applied electrical field—this is the inverse piezoelectric effect. The relationship between the direct and inverse effects is expressed as follows [66]:

$$\left\{\begin{array}{c} X=ST+dE\\ D=dT+\varepsilon E \end{array}\right.$$

where *S*—elastic compliance coefficient, *T*—stretching stress, *d*—piezoelectric coefficient, *E*—electric field, and ε—permittivity of the material.

Key parameters that could be considered significant in actuation applications of the piezomaterial are mainly as follows: electromechanical coupling coefficients (k_p_, k_15_, k_33_, k_31_, k_t_), piezoelectric modules (d_31_, d_33_, d_15_), longitudinal ultrasonic wave velocities (V^E^_1_, V^D^_4_, V^D^_3_), relative permittivity (ε^T^_33_/ε_0_ and ε^T^_11_/ε_0_), electrical capacitance, and resonance/antiresonance frequencies.

### 2.2. Piezoceramic Material Development and Investigation

PZT ceramics is a unique ferroelectric system, where “smeared” phase transitions can be realized in order to increase the morphotropic region [67]. Such systems are called ferroelectric relaxors [68]. By doping the PZT system with combined additives of lead magnoniobate, which in turn is a prominent representative of ferroelectric relaxors [69], as well as barium–strontium titanate, it was possible to obtain a new material: PKP-12 with a “smeared” phase transition. It has been established that the unique properties of relaxors are due to the formation and growth of nanoregions (nanodomains) in a crystal due to disorder in the environment of different ions located in crystallographically equivalent positions [70]. From the point of view of the use of these materials in the creation of multilayer actuators for wavefront correctors of the piezoactuator type, they make it possible to accurately control some properties of piezoceramics. For example, the value of the longitudinal piezoceramic coefficient d_33_ can be increased, which will lead to an increase in the displacement amplitude [65], however, this results in a decrease in the value of the Curie temperature.

The manufacturing of piezomaterials could be exploited two widely known ways: conventional [71] and colloidal-based techniques [72]. The main goal of both techniques is to obtain a homogeneous mixture of initial components—oxides or metals (Pb, Zr, Ti, etc.)

**Figure 2:**
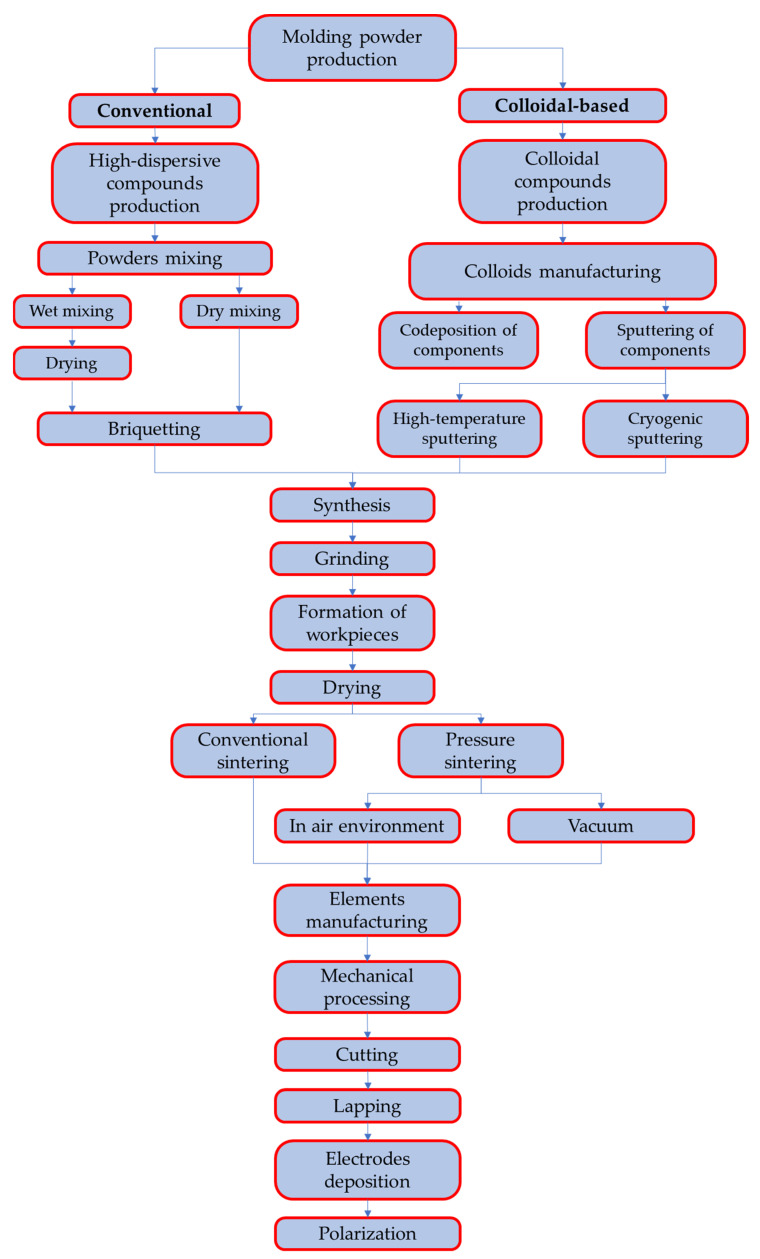
Scheme of piezoceramic material manufacturing process.

The main steps of these processes are shown in Figure 2 and it follows:

- For conventional ceramic technology, components are mixed in dry and wet grinding mills of a drum or planetary type.
- For colloidal-based method, the codeposition of components is carried out using a chemical reactor, centrifugation, and sputtering apparatuses.

After obtaining a homogeneous mechanical mixture, the synthesis stage occurs. Synthesis with a method using solid-state reactions is uniform heating (with a constant rate of temperature rise) to a target temperature of 800–900 degrees, at which the synthesis reaction of a compound with necessary composition occurs.

The synthesized compound is re-grinded in a drum or planetary mill, dried to a residual moisture content of no more than 0.2 percent, after which the powder is ready for formation.

Formation is carried out by uniaxial isostatic hot pressing (with preliminary plasticization in a spray dryer, or the addition of a plasticizer). Such method of manufacturing of the piezomaterial allows for obtaining the samples with high density and reduced quantity of the inner hollows that decrease the probability of the electrical breakdown and increase the local deformation of the piezoelements [73].

To determine the main parameters of the material, a number of samples were made using conventional ceramic technology. Silver electrodes were deposited on the surface of all developed samples, except for elements with longitudinal polarization, by firing a silver-containing paste. When applying an electric field of 8 kV/cm for 10 min at a temperature of 120 °C, followed by direct natural cooling to room temperature, the samples were polarized in air.

To obtain samples with longitudinal polarization, the sintered block was divided into separate elements with electrodes deposited by chemical deposition [74].

Using dynamic measurement methods, the main electromechanical properties of the material were studied: relative permittivity, dielectric loss tangent tgδ, electromechanical coupling coefficients, piezoelectric modules, ultrasonic waves velocities, mechanical quality factor Q_m_, Poisson’s ratio δp, and density ρ.

To obtain the electrical capacitance, resonance frequency, and antiresonance of the polarized piezoceramic samples, a Wayne Kerr Electronics WK 6510B precision impedance meter was used.

**Figure 3:**
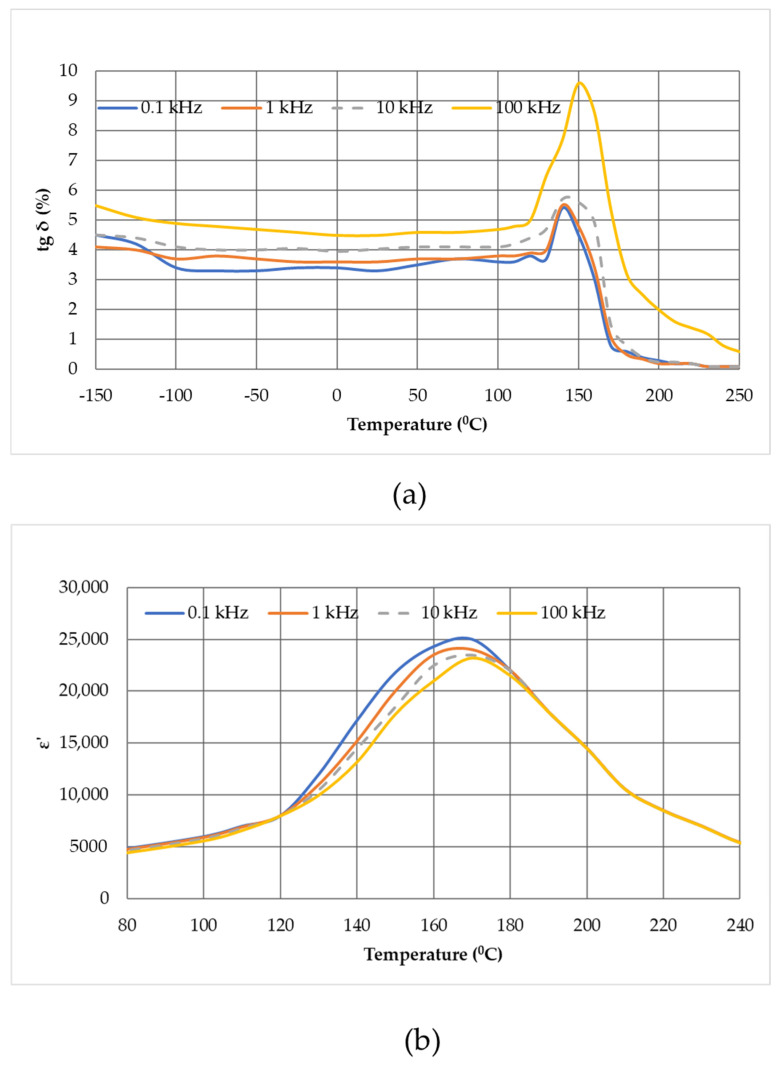
Dependence of the dielectric loss tangent *tgδ* (*T*) (**a**) and the relative permittivity *ε*′ (*T*) (**b**), and of the unpolarized composition under study from the temperature and frequency.

Figure 3 shows the temperature–frequency dependences of the relative permittivity (ε′ = ε⁄ε_0_) where ε is the permittivity of the material, ε_0_ is the dielectric constant, and tgδ is the dielectric loss tangent of unpolarized ceramics at frequencies of 0.1, 1, 10, and 100 kHz.

In the frequency range under consideration, a local temperature shift is clearly observed, where a maximum of the permittivity (T_m_) is observed as the frequency changes (Figure 3a), which is explicitly characteristic of relaxor ferroelectrics. In addition, the appearance of the dispression effect is observed (situation when curves start to split at the various frequencies) at a temperature significantly below T_m_. The maximum dispression of the permittivity is observed at a temperature of about 130 °C. It should also be noted that the maximum of the dielectric loss tangent tgδ (T) has an asymmetric form at different temperatures (Figure 3b), which is also a distinctive feature of ferroelectric materials with a diffuse phase transition [75].

**Figure 4:**
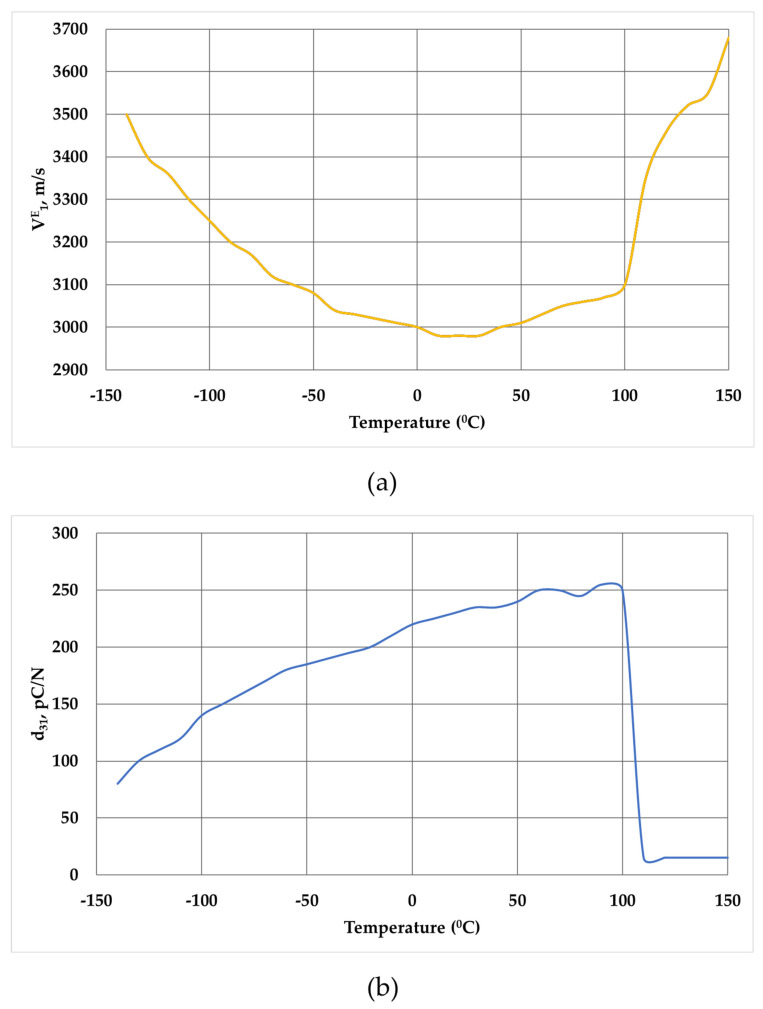
Temperature dependence of the longitudinal ultrasonic waves velocity V^E^_1_ (T) (**a**) and piezomodulus d_31_ (T) (**b**) of the developed material.

In our case, the possibility of the existence of a relaxor state in the material under study is also confirmed by the behavior of the elastic properties (Figure 4). So, from the temperature dependences of the piezomodulus d_31_ (T) and the velocity of the longitudinal sound wave V^E^_1_ (T) in a polarized sample, it follows that the anomalies d_31_ (T) in the form of a plateau-like maximum and V^E^_1_ (T) in the form of a very smeared minima are located significantly below T_m_. In this case, a sharp drop in the values of d_31_ (T) at T = 120 °C and a jump in V^E^_1_ (T) at T~110 °C are associated with the onset of depolarization of the sample.

Taking into account that the temperature of the velocity minimum both in classical ferroelectrics and in relaxor ferroelectrics [76] corresponds to a phase transition, the temperature range from 20 to 150 °C can be the region of phase coexistence. In the material under study, this can first be the region of coexistence of the low-temperature rhombohedral phase R3c and the high-temperature rhombohedral phase R3m, and then the region of coexistence of the rhombohedral and tetragonal phases, and in the T_m_ region of the tetragonal and cubic phases.

**Figure 5:**
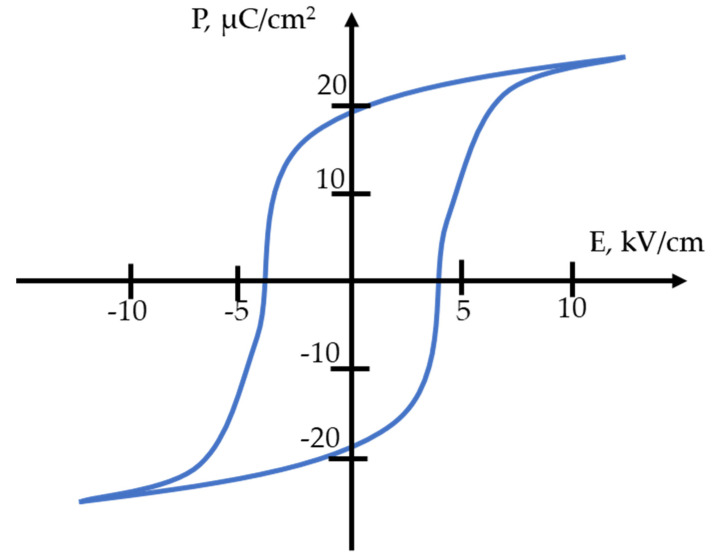
Hysteresis loop of the developed material.

The behavior of the polarization of the resulting material was also studied depending on the applied external field. Figure 5 shows the dielectric hysteresis loop of the material under study, from which the value of the coercive field E_C_ ≈ 4 kV/cm was determined. The hysteresis loop is saturated and close in shape to the loop characteristic of ferrosoft ceramics.

The values of elastic compliance coefficients were also obtained to be used for the numerical modeling of piezoelements created on the basis of this material. Elastic compliance determines the amount of deformation that occurs under the influence of applied mechanical stress (m^2^/N).

$$\left(\begin{array}{cccccc} S_{11}^{E} & S_{12}^{E} & S_{13}^{E} & 0 & 0 & 0\\ S_{21}^{E} & S_{22}^{E} & S_{23}^{E} & 0 & 0 & 0\\ S_{31}^{E} & S_{32}^{E} & S_{33}^{E} & 0 & 0 & 0\\ 0 & 0 & 0 & S_{44}^{E} & 0 & 0\\ 0 & 0 & 0 & 0 & S_{55}^{E} & 0\\ 0 & 0 & 0 & 0 & 0 & S_{66}^{E} \end{array}\right)=\left(\begin{array}{cccccc} 1.76 & -0.59 & -0.81 & 0 & 0 & 0\\ -0.59 & 1.76 & -0.81 & 0 & 0 & 0\\ -0.81 & -0.81 & 1.88 & 0 & 0 & 0\\ 0 & 0 & 0 & 4.47 & 0 & 0\\ 0 & 0 & 0 & 0 & 4.47 & 0\\ 0 & 0 & 0 & 0 & 0 & 4.71 \end{array}\right)\cdot 10^{-11}\;m^{2}/N$$

$$\left(\begin{array}{cccccc} S_{11}^{D} & S_{12}^{D} & S_{13}^{D} & 0 & 0 & 0\\ S_{21}^{D} & S_{22}^{D} & S_{23}^{D} & 0 & 0 & 0\\ S_{31}^{D} & S_{32}^{D} & S_{33}^{D} & 0 & 0 & 0\\ 0 & 0 & 0 & S_{44}^{D} & 0 & 0\\ 0 & 0 & 0 & 0 & S_{55}^{D} & 0\\ 0 & 0 & 0 & 0 & 0 & S_{66}^{D} \end{array}\right)=\left(\begin{array}{cccccc} 1.5 & -0.85 & -0.32 & 0 & 0 & 0\\ -0.85 & 1.5 & -0.32 & 0 & 0 & 0\\ -0.32 & -0.32 & 0.94 & 0 & 0 & 0\\ 0 & 0 & 0 & 2.21 & 0 & 0\\ 0 & 0 & 0 & 0 & 2.21 & 0\\ 0 & 0 & 0 & 0 & 0 & 4.71 \end{array}\right)\cdot 10^{-11}\;m^{2}/N$$

Here, $S_{ij}^{E}$ is the elastic compliance for stress in direction *i* (perpendicular to the direction in which the ceramic element is polarized) and accompanying strain in direction *j*, under a constant electric field (short circuit), and $S_{ij}^{D}$ is the elastic compliance for stress in direction *i* (parallel to direction in which the ceramic element is polarized) and accompanying strain in direction *j*, under constant electric displacement (open circuit).

After that, the electrophysical characteristics of this composition were measured under standard conditions (T = 20 ± 5 °C). The measured data are presented in Table 1. Based on the data obtained, it can be established that the material under study has high values of dielectric permittivity, piezoelectric coefficients, and electromechanical coupling coefficients, which ultimately allow for the use of this material in the manufacturing of the devices for the tasks of actuator technology. Moreover, in comparison with piezomaterials from other manufacturers, this ceramic has a high value of the piezoelectric coefficient d_33_ and, at the same time, a low value of the dielectric loss tangent. A comparative analysis of the key parameters of widely known piezoelectric materials is shown in Table 2.

## 3. Results and Discussion

**Figure 6:**
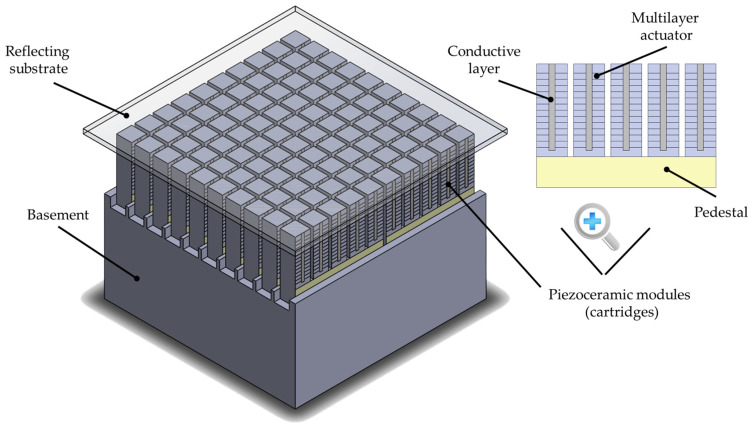
Scheme of the piezostack deformable mirror with modular design.

First of all, it is worth mentioning the principle of operation of the piezostack deformable mirror with modular design. In fact, this mirror is one of the modifications of a piezoactuator deformable mirror and consists of a relatively thin reflective plate, under which there are modules (cartridges) with individual multilayer piezoelectric stacks, assembled on a single pedestal and glued at one end to the rear side of the passive substrate, others—to a thick basement (Figure 6). Due to the inverse piezoelectric effect, when a control voltage is applied, the length of the piezostack changes. Since the piezoelectric actuator is rigidly fixed on the back side of the substrate, its elongation causes local deformation of the reflecting surface. By applying a set of different voltages to such control elements, it is possible to form a desirable arbitrary shape of the surface of a deformable mirror.

Based on the research data on the characteristics of piezomaterials presented in Section 2, multilayer modules with five separate control elements were made, although it is possible to create such modules with a large number of actuators, nevertheless, to ensure versatility and create deformable mirrors.

**Figure 7:**
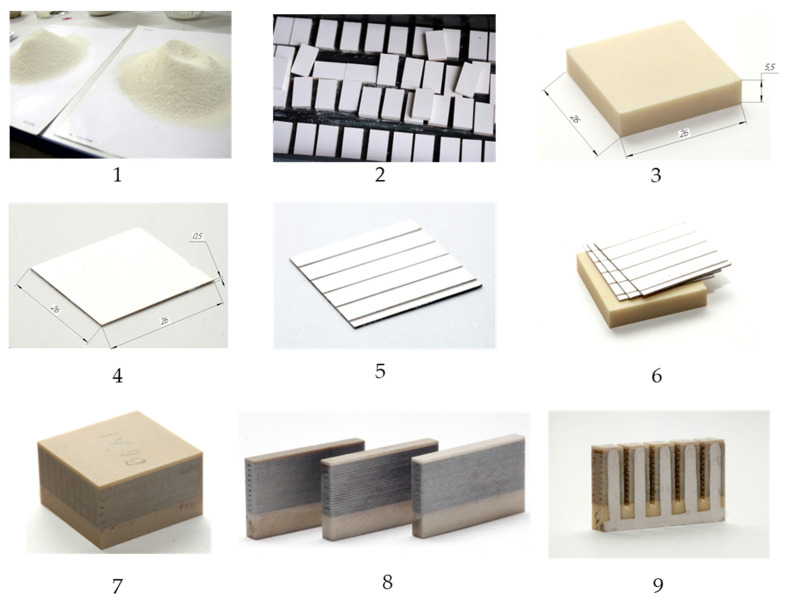
Scheme of the production cycle of the piezostack modules manufacturing.

The production process of multi-element piezoceramic actuators is shown in Figure 7 and consists of the following steps:

First, a mixture (mixture of starting materials) of the PKP-12 piezoceramic material was prepared using the classical ceramic technology from metal oxides. The material was synthesized by the method of solid-phase reactions by firing the mixture in a muffle furnace at a temperature of 850 °C. After that, the mixture obtained was crushed into powder and granulated (1).

Then, the PKP-12 granular material was pressed into blocks, which were subsequently dried in an oven and fired in a muffle furnace at a temperature of 1230 °C (2). The sintered blocks were ground on all sides, then sawn into 5-mm-thick base elements (3) and 0.5-mm-thick active layers (4).

After that, a silver-containing paste was applied to the active piezoelectric layers, which was fired by soft annealing at a temperature of up to 700 °C. Next, part of the silver coating was removed in order to separate the tracks (5).

Prepared base elements sized 26 × 26 × 5.5 mm were assembled into a package with active layers (26 × 26 × 0.5 mm), after which the compression welding of the piezoceramic block (6) took place. The assembled and welded block was again subjected to mechanical processing (grinding of the end surfaces) (7), after which it was sawn into separate modules (8).

Further, the cut parts of the block were subjected to further sawing into separate actuators in such a way that the active layers were separated from each other by a gap of 1 mm, but remained rigidly fixed to the base element (9).

**Figure 8:**
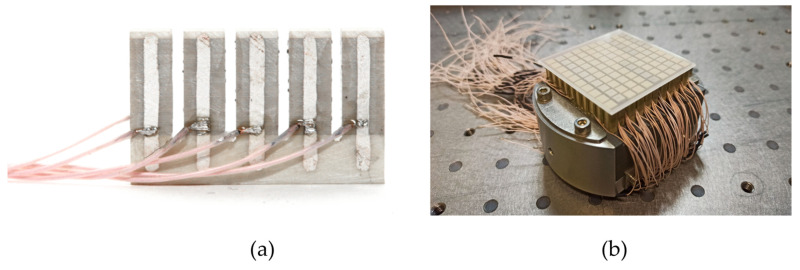
Piezostack deformable mirror with modular design: piezoceramic module with wiring (**a**), main view of the assembled deformable mirror (**b**).

Each actuator of the resulting module was covered with a layer of silver-containing paste (burning method) in order to switch the electrodes of the active layers. Then, an SiF wire 0.4 mm thick was soldered to each current lead (Figure 8a).

Next, the process of polarization of the piezoceramics was carried out and the characteristics of individual actuators in the module were measured.

The obtained values are given in Table 3.

It should be noted that the developed modules have a surface deformation amplitude of up to 4.3 µm. Taking into account the fact that part of the range fails in overcoming the elastic forces due to the deformation of the reflecting substrate, in a piezoactuator deformable mirror, the local displacement will decrease by about 50% and will be >2 μm. Thus, these elements can be used in deformable mirrors of the piezoactuator type to correct for the phase fluctuations of the atmosphere of the Kolmogorov spectrum [77] on a path 1500 m long, a beam diameter of 250 mm, a structural constant of the refractive index of C_n_^2^ = 10^–14^ m^–2/3^, and a coherence radius of 71 mm at a wavelength of 1064 nm [78,79,80].

In addition, when determining the speed of the system, one of the important indicators of the response speed will be the capacity of the control elements. Since the control element of actuator-type deformable mirrors is actually a piezoelectric capacitor, to control the charge and discharge of this capacitor, it is necessary to use the concept of an RC circuit, which shows the time during which the piezoceramic element has time to charge/discharge by 63% [81] of the initial voltage applied to change the length of the piezoplate or piezostack (the magnitude of the applied voltage does not affect the charge rate of the capacitor). Therefore, from the point of view of control theory, it is reasonable to use deformable mirrors with a lower value of the capacitance of the control elements. The values of electric capacitance for conventional mirrors are in the range up to 0.5 μF [22,23,24,25,26,27,28,29,30,31].

In order to manufacture a piezostack deformable mirror with modular design with matrix of elements 10 × 10, we combined the cartridges on the rigid basement made of piezoceramic material. An aperture of the mirror of 50 x50 mm was chosen with square geometry of the control elements, however, such method of assembling allows for acquiring any net of the actuators and to scale the aperture of the deformable mirror. After assembly of the piezomodule part, the thin polished 1.5 mm glass substrate was glued on this part using the reference plate (λ/4) to obtain a high quality of the initial surface of the mirror (Figure 8b).

After development of the modular piezostack deformable mirror, we investigated the key parameters of such a wavefront corrector–response function of actuators, and the amplitude-phase characteristic.

**Figure 9:**
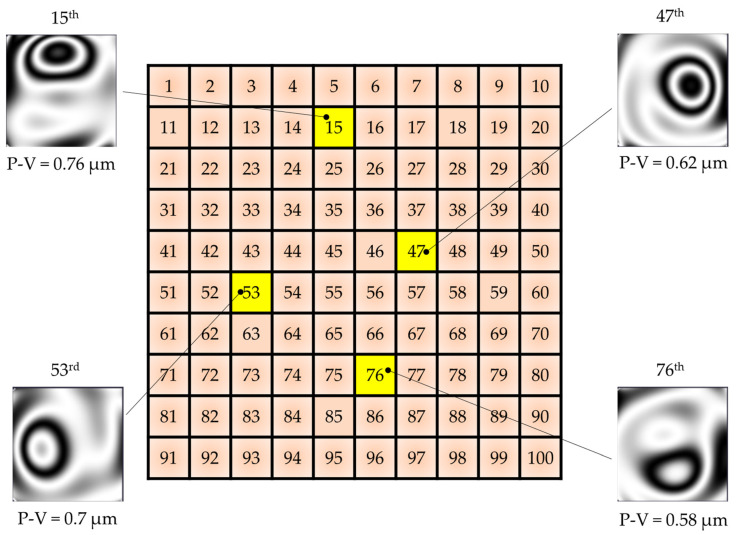
Response functions of the 15th, 47th, 53rd, and 76th actuators of the modular piezostack deformable mirror.

To measure the response functions of the control elements of the developed deformable mirror, the diagnostic setup based on the Shack–Hartmann wavefront sensor was used. The images of the mirror surface after applying the voltage of 300 V to different actuators are presented in Figure 9. As it could be seen from the presented data, the main surface deformation is of course concentrated in the range of the position of the actuators. At the same time, on the neighbor actuators one can also see the change in the mirror surface. This is due to the small (only 1 mm) distance between the actuators and also rather high thickness of the reflecting substrate (1.5 mm). In our case, the coupling coefficient was in the range of 60%. In addition, we expected the local stroke of the developed deformable mirror actuators be close to two microns, however, the measured data showed that the real local mirror displacement was just about one micron. Since the thickness of the individual layer of the actuator is 500 μm, the control voltages could be increased up to 600 V (−200…+400 V) for the manufactured piezoceramic modules. Thus, the stroke should be close to the expected 2 μm.

**Figure 10:**
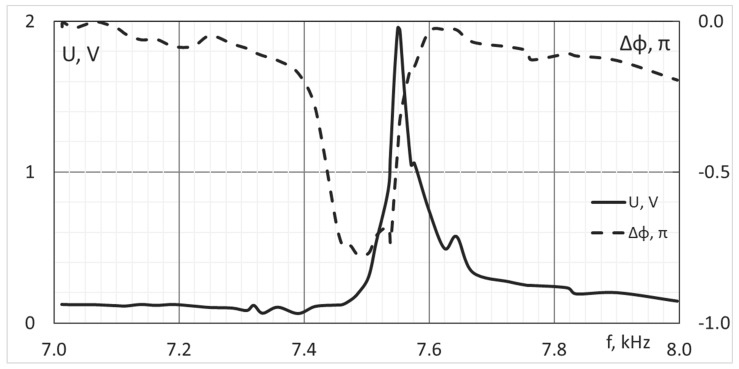
Amplitude-frequency (solid line) and phased-frequency (dashed line) responses of the developed piezostack deformable mirror.

In addition, the first resonant frequency of the mirror was measured (Figure 10) by measuring amplitude-frequency and phased-frequency responses of the mirror. To measure the real value of this parameter we used the oscilloscope Tektronix DPO-2004B and a sine wave generator. The sine voltage with the frequency range from 0.1 to 8 kHz was applied to the actuator #45 (it could be applied to any piezostack). Due to the piezoelectric effect, AC voltage was detected on the neighbor electrode #46 and was registered using the oscilloscope. The amplitude of the output signal was stable up to the frequency of 7.4 kHz, and the first resonance was found at the frequency of 7.5 kHz. Additionally, the temporal bandwidth of the deformable mirror could be described using the Bode Curve [82], which shows the use of such corrector in an adaptive optical system with frequency up to 7.4 kHz.

## 4. Conclusions

Based on the PZT ceramic, the PKP-12 piezoceramic material was produced. Key properties of the developed material such as electromechanical coupling coefficients (k_p_, k_15_, k_33_, k_31_, k_t_), piezoelectric modules (d_31_, d_33_, d_15_), longitudinal ultrasonic waves velocities (V^E^_1_, V^D^_4_, V^D^_3_), relative permittivity (ε^T^_33_/ε_0_ and ε^T^_11_/ε_0_), electrical capacitance, resonance/antiresonance frequencies, dielectric loss tangent, mechanical quality factor, Poisson’s ratio, and density were investigated. It was shown that in terms of elastic properties this material is very close to ferroelectric relaxors, having high compliance in a wide temperature range. In addition, the resulting material had a high value of the longitudinal piezoceramic coefficient of 660 pC/N and a low value of the dielectric loss tangent (no more than 1.5%). Therefore, such material could be applied for manufacturing of the multilayer actuators.

Based on the study of the electrophysical and mechanical parameters of the obtained materials, piezoceramic modules were fabricated with five multilayer actuators 4 × 4 × 15 mm. The capacitance of the individual actuator was 12 nF. The maximum displacement at a control voltage of 300 V was in the range of 4.1–4.3 µm. The developed piezoelectric elements were used for the creation of the piezostack deformable mirror with modular design with the possibility of obtaining the required configuration of control elements by combining the developed piezoceramic modules.

## Figures and Tables

**Table 1:** Electrophysical characteristics of the developed material.

Parameter	Value
Curie temperature, T_c_	160 °C
Relative permittivity ε^T^_33_/ε_0_	4950
Relative permittivity ε^T^_11_/ε_0_	4400
Dielectric loss tangent tgδ,	1.5%
Electromechanical coupling coefficient k_p_	0.66
Electromechanical coupling coefficient k_15_	0.71
Electromechanical coupling coefficient k_33_	0.70
Electromechanical coupling coefficient k_31_	0.38
Electromechanical coupling coefficient k_t_	0.53
Piezoelectrical coefficient |d_31_|	325 × 10^−12^ C/N
Piezoelectrical coefficient d_33_	660 × 10^−12^ C/N
Piezoelectrical coefficient d_15_	940 × 10^−12^ C/N
Longitudinal ultrasonic waves velocity V^E^_1_	2770 × 10^3^ m/s
Longitudinal ultrasonic waves velocity V^D^_4_	2470 × 10^3^ m/s
Longitudinal ultrasonic waves velocity V^D^_3_	3700 × 10^3^ m/s
Quality factor Q_m_	60
Poisson ratio δp	0.34
Density ρ	7400 kg/m^3^

**Table 2:** Electrophysical characteristics of the developed material in comparison with piezoceramics available in the market.

Parameter	PKP-12	Sonox P51 (CeramTec AG, Plochingen, Germany)	OPT 5100 (Omega Piezo Technologies Inc., State College, PA, USA)	N-10 (Tokin, Shiroishi, Japan)
Relative permittivity ε^T^_33_/ε_0_	4950	3100	3200	5440
Dielectric loss tangent tgδ, %	1.5	16	2	2
Piezoelectrical coefficient |d_31_| (10^−12^), C/N	325	260	274	287
Piezoelectrical coefficient d_33_ (10^−12^), C/N	660	640	550	635
Piezoelectrical coefficient d_15_ (10^−12^), C/N	940	730	780	930

**Table 3:** Parameters of the manufactured piezomodules.

Parameter	Value
Capacitance of the single piezostack, C_nom_	11–12 nF
Stroke under 300 V	4.1–4.3 µ

## Data Availability

The data that supports the findings of this study are available from the corresponding author upon reasonable request.
